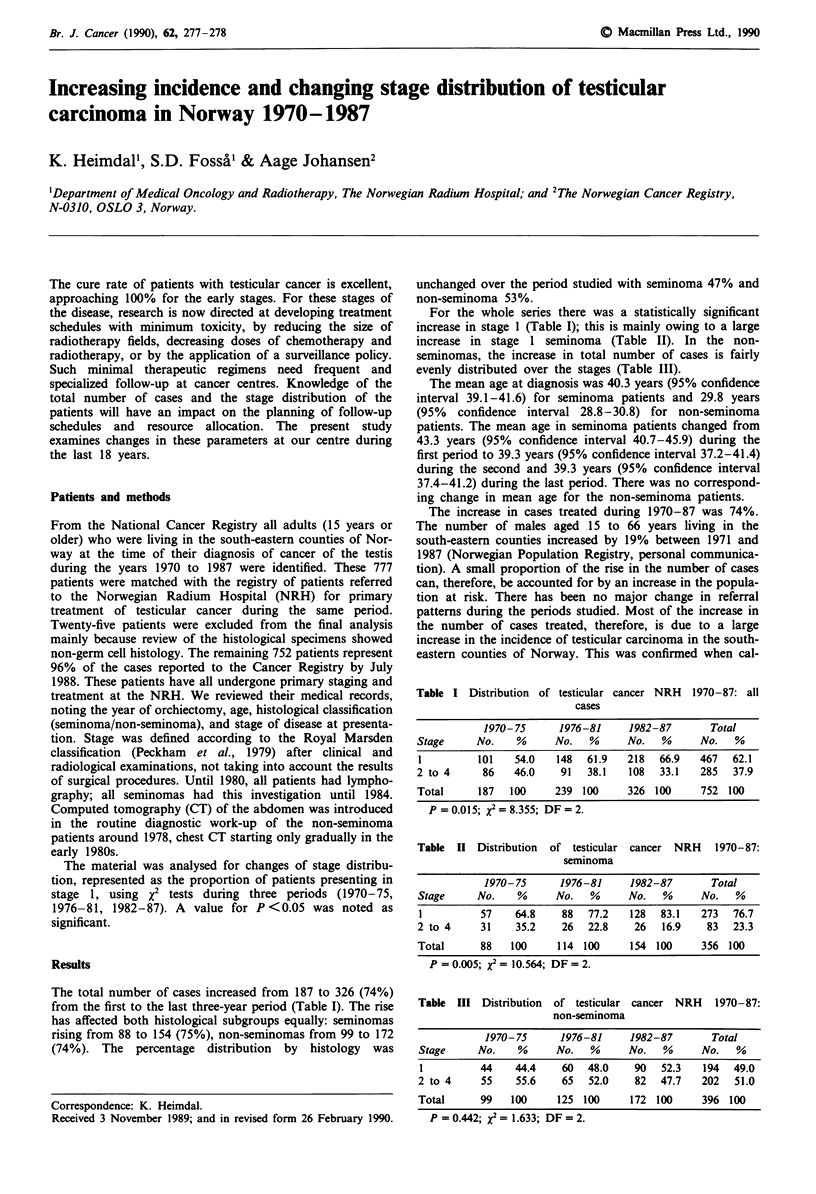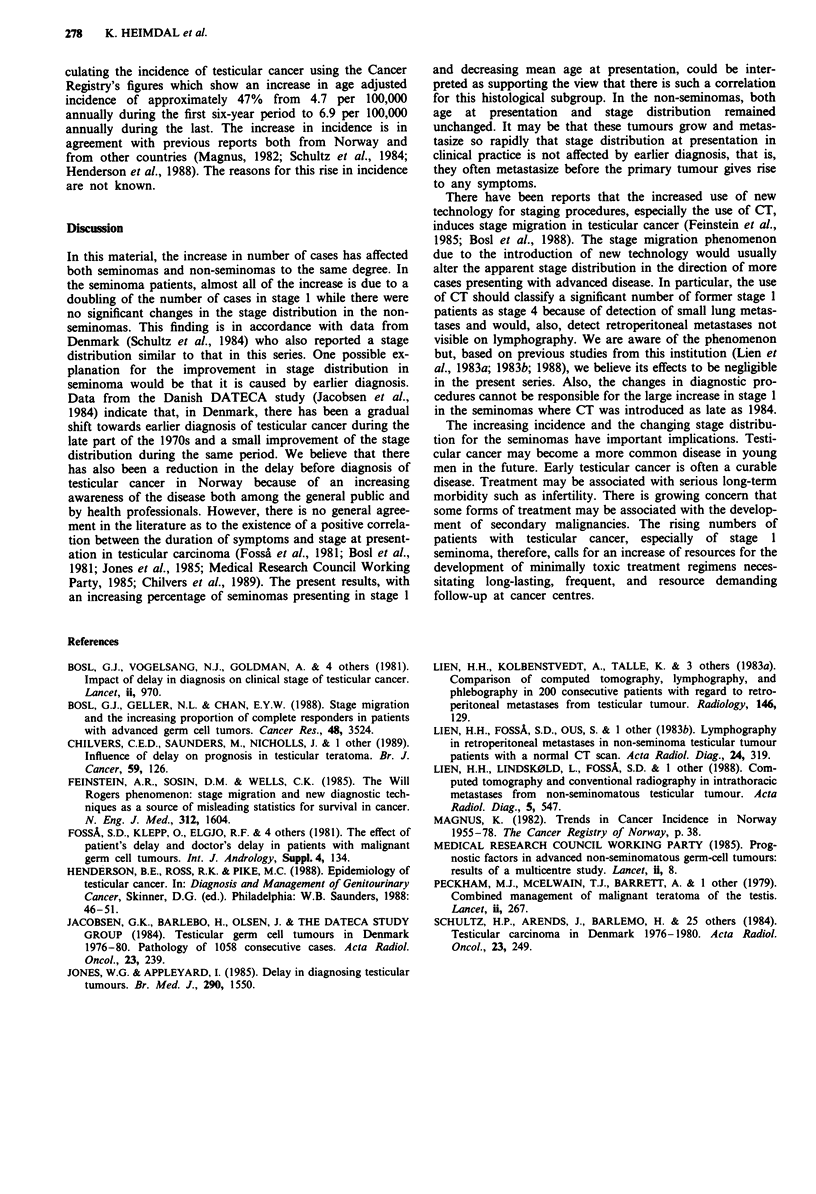# Increasing incidence and changing stage distribution of testicular carcinoma in Norway 1970-1987.

**DOI:** 10.1038/bjc.1990.277

**Published:** 1990-08

**Authors:** K. Heimdal, S. D. Fosså, A. Johansen

**Affiliations:** Department of Medical Oncology and Radiotherapy, Norwegian Radium Hospital, Oslo.


Br. J. Cancer (1990), 62, 277-278 © Macmillan Press Ltd., 1990

### Increasing incidence and changing stage distribution of testicular carcinoma in Norway 1970-1987

K. Heimdal[1], S.D. Fosså[1] & Aage Johansen[2]

[1]*Department of Medical Oncology and Radiotherapy, The Norwegian Radium Hospital; and* [2]*The Norwegian Cancer Registry, N-0310, OSLO 3, Norway.*

The cure rate of patients with testicular cancer is excellent, approaching 100% for the early stages. For these stages of the disease, research is now directed at developing treatment schedules with minimum toxicity, by reducing the size of radiotherapy fields, decreasing doses of chemotherapy and radiotherapy, or by the application of a surveillance policy. Such minimal therapeutic regimens need frequent and specialized follow-up at cancer centres. Knowledge of the total number of cases and the stage distribution of the patients will have an impact on the planning of follow-up schedules and resource allocation. The present study examines changes in these parameters at our centre during the last 18 years.

### Patients and methods

From the National Cancer Registry all adults (15 years or older) who were living in the south-eastern counties of Norway at the time of their diagnosis of cancer of the testis during the years 1970 to 1987 were identified. These 777 patients were matched with the registry of patients referred to the Norwegian Radium Hospital (NRH) for primary treatment of testicular cancer during the same period. Twenty-five patients were excluded from the final analysis mainly because review of the histological specimens showed non-germ cell histology. The remaining 752 patients represent 96% of the cases reported to the Cancer Registry by July 1988. These patients have all undergone primary staging and treatment at the NRH. We reviewed their medical records, noting the year of orchiectomy, age, histological classification (seminoma/non-seminoma), and stage of disease at presentation. Stage was defined according to the Royal Marsden classification (Peckham *et al.*, 1979) after clinical and radiological examinations, not taking into account the results of surgical procedures. Until 1980, all patients had lymphography; all seminomas had this investigation until 1984. Computed tomography (CT) of the abdomen was introduced in the routine diagnostic work-up of the non-seminoma patients around 1978, chest CT starting only gradually in the early 1980s.

The material was analysed for changes of stage distribution, represented as the proportion of patients presenting in stage 1, using $\chi^2$ tests during three periods (1970-75, 1976-81, 1982-87). A value for $P < 0.05$ was noted as significant.

### Results

The total number of cases increased from 187 to 326 (74%) from the first to the last three-year period (Table I). The rise has affected both histological subgroups equally: seminomas rising from 88 to 154 (75%), non-seminomas from 99 to 172 (74%). The percentage distribution by histology was unchanged over the period studied with seminoma 47% and non-seminoma 53%.

For the whole series there was a statistically significant increase in stage 1 (Table I); this is mainly owing to a large increase in stage 1 seminoma (Table II). In the non-seminomas, the increase in total number of cases is fairly evenly distributed over the stages (Table III).

The mean age at diagnosis was 40.3 years (95% confidence interval 39.1-41.6) for seminoma patients and 29.8 years (95% confidence interval 28.8-30.8) for non-seminoma patients. The mean age in seminoma patients changed from 43.3 years (95% confidence interval 40.7-45.9) during the first period to 39.3 years (95% confidence interval 37.2-41.4) during the second and 39.3 years (95% confidence interval 37.4-41.2) during the last period. There was no corresponding change in mean age for the non-seminoma patients.

The increase in cases treated during 1970-87 was 74%. The number of males aged 15 to 66 years living in the south-eastern counties increased by 19% between 1971 and 1987 (Norwegian Population Registry, personal communication). A small proportion of the rise in the number of cases can, therefore, be accounted for by an increase in the population at risk. There has been no major change in referral patterns during the periods studied. Most of the increase in the number of cases treated, therefore, is due to a large increase in the incidence of testicular carcinoma in the south-eastern counties of Norway. This was confirmed when cal-

**Table I** Distribution of testicular cancer NRH 1970-87: all cases

| | *1970-75* | | *1976-81* | | *1982-87* | | *Total* | |
|---|---|---|---|---|---|---|---|---|
| *Stage* | *No.* | *%* | *No.* | *%* | *No.* | *%* | *No.* | *%* |
| 1 | 101 | 54.0 | 148 | 61.9 | 218 | 66.9 | 467 | 62.1 |
| 2 to 4 | 86 | 46.0 | 91 | 38.1 | 108 | 33.1 | 285 | 37.9 |
| Total | 187 | 100 | 239 | 100 | 326 | 100 | 752 | 100 |

$P = 0.015$; $\chi^2 = 8.355$; DF = 2.

**Table II** Distribution of testicular cancer NRH 1970-87: seminoma

| | *1970-75* | | *1976-81* | | *1982-87* | | *Total* | |
|---|---|---|---|---|---|---|---|---|
| *Stage* | *No.* | *%* | *No.* | *%* | *No.* | *%* | *No.* | *%* |
| 1 | 57 | 64.8 | 88 | 77.2 | 128 | 83.1 | 273 | 76.7 |
| 2 to 4 | 31 | 35.2 | 26 | 22.8 | 26 | 16.9 | 83 | 23.3 |
| Total | 88 | 100 | 114 | 100 | 154 | 100 | 356 | 100 |

$P = 0.005$; $\chi^2 = 10.564$; DF = 2.

**Table III** Distribution of testicular cancer NRH 1970-87: non-seminoma

| | *1970-75* | | *1976-81* | | *1982-87* | | *Total* | |
|---|---|---|---|---|---|---|---|---|
| *Stage* | *No.* | *%* | *No.* | *%* | *No.* | *%* | *No.* | *%* |
| 1 | 44 | 44.4 | 60 | 48.0 | 90 | 52.3 | 194 | 49.0 |
| 2 to 4 | 55 | 55.6 | 65 | 52.0 | 82 | 47.7 | 202 | 51.0 |
| Total | 99 | 100 | 125 | 100 | 172 | 100 | 396 | 100 |

$P = 0.442$; $\chi^2 = 1.633$; DF = 2.

Correspondence: K. Heimdal.
Received 3 November 1989; and in revised form 26 February 1990.

culating the incidence of testicular cancer using the Cancer Registry's figures which show an increase in age adjusted incidence of approximately 47% from 4.7 per 100,000 annually during the first six-year period to 6.9 per 100,000 annually during the last. The increase in incidence is in agreement with previous reports both from Norway and from other countries (Magnus, 1982; Schultz *et al.*, 1984; Henderson *et al.*, 1988). The reasons for this rise in incidence are not known.

### Discussion

In this material, the increase in number of cases has affected both seminomas and non-seminomas to the same degree. In the seminoma patients, almost all of the increase is due to a doubling of the number of cases in stage 1 while there were no significant changes in the stage distribution in the non-seminomas. This finding is in accordance with data from Denmark (Schultz *et al.*, 1984) who also reported a stage distribution similar to that in this series. One possible explanation for the improvement in stage distribution in seminoma would be that it is caused by earlier diagnosis. Data from the Danish DATECA study (Jacobsen *et al.*, 1984) indicate that, in Denmark, there has been a gradual shift towards earlier diagnosis of testicular cancer during the late part of the 1970s and a small improvement of the stage distribution during the same period. We believe that there has also been a reduction in the delay before diagnosis of testicular cancer in Norway because of an increasing awareness of the disease both among the general public and by health professionals. However, there is no general agreement in the literature as to the existence of a positive correlation between the duration of symptoms and stage at presentation in testicular carcinoma (Fosså *et al.*, 1981; Bosl *et al.*, 1981; Jones *et al.*, 1985; Medical Research Council Working Party, 1985; Chilvers *et al.*, 1989). The present results, with an increasing percentage of seminomas presenting in stage 1 and decreasing mean age at presentation, could be interpreted as supporting the view that there is such a correlation for this histological subgroup. In the non-seminomas, both age at presentation and stage distribution remained unchanged. It may be that these tumours grow and metastasize so rapidly that stage distribution at presentation in clinical practice is not affected by earlier diagnosis, that is, they often metastasize before the primary tumour gives rise to any symptoms.

There have been reports that the increased use of new technology for staging procedures, especially the use of CT, induces stage migration in testicular cancer (Feinstein *et al.*, 1985; Bosl *et al.*, 1988). The stage migration phenomenon due to the introduction of new technology would usually alter the apparent stage distribution in the direction of more cases presenting with advanced disease. In particular, the use of CT should classify a significant number of former stage 1 patients as stage 4 because of detection of small lung metastases and would, also, detect retroperitoneal metastases not visible on lymphography. We are aware of the phenomenon but, based on previous studies from this institution (Lien *et al.*, 1983*a*; 1983*b*; 1988), we believe its effects to be negligible in the present series. Also, the changes in diagnostic procedures cannot be responsible for the large increase in stage 1 in the seminomas where CT was introduced as late as 1984.

The increasing incidence and the changing stage distribution for the seminomas have important implications. Testicular cancer may become a more common disease in young men in the future. Early testicular cancer is often a curable disease. Treatment may be associated with serious long-term morbidity such as infertility. There is growing concern that some forms of treatment may be associated with the development of secondary malignancies. The rising numbers of patients with testicular cancer, especially of stage 1 seminoma, therefore, calls for an increase of resources for the development of minimally toxic treatment regimens necessitating long-lasting, frequent, and resource demanding follow-up at cancer centres.

### References

BOSL, G.J., VOGELSANG, N.J., GOLDMAN, A. & 4 others (1981). Impact of delay in diagnosis on clinical stage of testicular cancer. *Lancet*, **ii**, 970.

BOSL, G.J., GELLER, N.L. & CHAN, E.Y.W. (1988). Stage migration and the increasing proportion of complete responders in patients with advanced germ cell tumors. *Cancer Res.*, **48**, 3524.

CHILVERS, C.E.D., SAUNDERS, M., NICHOLLS, J. & 1 other (1989). Influence of delay on prognosis in testicular teratoma. *Br. J. Cancer*, **59**, 126.

FEINSTEIN, A.R., SOSIN, D.M. & WELLS, C.K. (1985). The Will Rogers phenomenon: stage migration and new diagnostic techniques as a source of misleading statistics for survival in cancer. *N. Eng. J. Med.*, **312**, 1604.

FOSSÅ, S.D., KLEPP, O., ELGJO, R.F. & 4 others (1981). The effect of patient's delay and doctor's delay in patients with malignant germ cell tumours. *Int. J. Andrology*, **Suppl. 4**, 134.

HENDERSON, B.E., ROSS, R.K. & PIKE, M.C. (1988). Epidemiology of testicular cancer. In: *Diagnosis and Management of Genitourinary Cancer*, Skinner, D.G. (ed.). Philadelphia: W.B. Saunders, 1988: 46-51.

JACOBSEN, G.K., BARLEBO, H., OLSEN, J. & THE DATECA STUDY GROUP (1984). Testicular germ cell tumours in Denmark 1976-80. Pathology of 1058 consecutive cases. *Acta Radiol. Oncol.*, **23**, 239.

JONES, W.G. & APPLEYARD, I. (1985). Delay in diagnosing testicular tumours. *Br. Med. J.*, **290**, 1550.

LIEN, H.H., KOLBENSTVEDT, A., TALLE, K. & 3 others (1983*a*). Comparison of computed tomography, lymphography, and phlebography in 200 consecutive patients with regard to retroperitoneal metastases from testicular tumour. *Radiology*, **146**, 129.

LIEN, H.H., FOSSÅ, S.D., OUS, S. & 1 other (1983*b*). Lymphography in retroperitoneal metastases in non-seminoma testicular tumour patients with a normal CT scan. *Acta Radiol. Diag.*, **24**, 319.

LIEN, H.H., LINDSKØLD, L., FOSSÅ, S.D. & 1 other (1988). Computed tomography and conventional radiography in intrathoracic metastases from non-seminomatous testicular tumour. *Acta Radiol. Diag.*, **5**, 547.

MAGNUS, K. (1982). Trends in Cancer Incidence in Norway 1955-78. *The Cancer Registry of Norway*, p. 38.

MEDICAL RESEARCH COUNCIL WORKING PARTY (1985). Prognostic factors in advanced non-seminomatous germ-cell tumours: results of a multicentre study. *Lancet*, **ii**, 8.

PECKHAM, M.J., MCELWAIN, T.J., BARRETT, A. & 1 other (1979). Combined management of malignant teratoma of the testis. *Lancet*, **ii**, 267.

SCHULTZ, H.P., ARENDS, J., BARLEMO, H. & 25 others (1984). Testicular carcinoma in Denmark 1976-1980. *Acta Radiol. Oncol.*, **23**, 249.